# COVID-19 in Europe: Dataset at a sub-national level

**DOI:** 10.1016/j.dib.2021.106939

**Published:** 2021-03-03

**Authors:** Hichem Omrani, Madalina Modroiu, Javier Lenzi, Bilel Omrani, Zied Said, Marc Suhrcke, Anastase Tchicaya, Nhien Nguyen, Benoit Parmentier

**Affiliations:** a Urban Development and Mobility, Luxembourg Institute of Socio-Economic Research, Luxembourg; b École Centrale de Lyon, France; c Polytechnique Montréal, Canada; d Norwegian University of Science and Technology, Norway; e University of Maryland, USA; f Centre for Health Economics, University of York, UK

**Keywords:** SARS-CoV-2 coronavirus disease, COVID-19 mortality, COVID-19 infections, Socioeconomic-demographic factors, Air pollution, Environment, Health, NUTS3, Europe

## Abstract

The COVID-19 pandemic has hit humanity, straining health care systems, economies, and governments worldwide. In one of the responses to the pandemic, a big global effort has been mounted to collect, analyze, and make data publicly available. However, many of the existing COVID-19 public datasets are (i) aggregated at country level, and (ii) tend not to bring the COVID-19-specific data coupled with socio-demographic, economic, public policy, health, pollution and environmental factors, all of which may be key elements to study the transmission of the SARS-CoV-2 and its severity. To aid the evaluation of the determinants and impact of the COVID-19 pandemic at a large scale, we present here a new dataset with socio-demographic, economic, public policy, health, pollution and environmental factors for the European Union at the small regions level (NUTS3). The database is freely accessible at http://dx.doi.org/10.17632/2ghxnrkr9p.4. This dataset can help to monitor the COVID-19 mortality and infections at the sub-national level and enable analysis that may inform future policymaking.

## Specifications Table

**Table:** 

Subject	Environmental science
Specific subject area	COVID-19, Health, Pollution, Environment, Climate, socioeconomic factors
Type of data	Table
How data were acquired	Open data sources from health statistics, governmental data, census data and public datasets, as well as satellite data for environmental, climate and environment.
Data format	Analyzed Filtered
Parameters for data collection	The dataset was constructed as a compilation of sub-national datasets at NUTS3 or NUTS2 (region level; in case not reported at NUTS3) for 18 European countries [1]. This dataset currently feeds our online COVID-19*-*transmission dashboard [2].
Description of data collection	The data were collected from open data sources for socio-demographics, economic, public policy, health, air pollution and environmental variables. The socio-demographic and economic data were gathered from the Eurostat website. Additionally, we collected nightlight intensity (NLI) data from the Defense Meteorological Satellite Program (DMSP) Operational Linescan System (OLS) and aggregated at the NUTS3 level. This NLI is a good proxy of Gross Domestic Product-GDP [3]. COVID-19 mortality, the number of positive cases, number of tests, as well as public policies, including the lag of lockdown implementations (i.e., number of days since the first case reported until the first day of lockdown), lockdown duration and severity were collected from open source repositories of each country (public dashboards, governmental health care ministries or agencies). Health data regarding the health status of the population (percentage of smokers, percentage of the population with chronic obstructive pulmonary disease, with diabetes) and mortality rates (i.e. deaths per 100.000 inhabitants resulting from respiratory diseases, cardiovascular disease, or diabetes) were collected from governmental open data source of each country at sub-national level (NUTS2). Air pollution data was collected from the Sentinel-5P satellite using Copernicus’ application programming interface (API). Data was downloaded at a resolution of 7 × 3.5 km, filtered, resampled, and aggregated at the NUTS3 level. Environmental datasets (temperature, solar radiation, humidity, precipitation, and wind speed) were collected from WorldClim Version 2 and Leaf Area index as a measure of greenness were collected from the NOAA Climate Data Record (CDR) of Advanced Very High Resolution Radiometer (AVHRR) Surface Reflectance. Both datasets were downloaded at a resolution of 30 s (~1.1 km^2^) and aggregated at NUTS3 level.
Data source location	Austria, Belgium, Denmark, France, Germany, Greece, Italy, Luxembourg, Netherlands, Norway, Poland, Portugal, Romania, Slovenia, Spain, Sweden, Switzerland and United Kingdom. For a full list of the sources by location refer to the “Sources” sheet of the dataset.
Data accessibility	Data is supplied on Mendeley (Public repository) Repository name: http://dx.doi.org/10.17632/2ghxnrkr9p.4

## Value of the Data

• This dataset is a useful input to improve the understanding of the inter-relationships between COVID-19 mortality and infections with socio-demographic, economic, public policy, health, air pollution and environmental factors at the finest possible level of spatial (NUTS2-3) and temporal (daily, weekly, monthly) resolutions  in fighting the pandemic across Europe.
• The beneficiaries of these data are the general public, policy-makers, organizations, researchers who deal with the COVID-19 spread from local (sub-country) to large scale (continental). These data can be used: (i) to conduct a cross-comparison between European countries either at NUTS2 or at NUTS3 level, (ii) to inform European citizens on the COVID-19 spread in Europe, and (iii) to support researchers in future socio-epidemiological research.
• It can be combined with survey or census health data for a wide range of applications. The dataset contributes to a better scientific understanding of the COVID-19 outbreak, to facilitate the process of searching for science-driven solutions.

## 1 Data Description

In Table 1, we present several key variables of this dataset: the health data regarding the COVID-19 cases, mortality, and tests performed at sub-national level (NUTS3), collected until August 31st 2020. Furthermore, we include in Table 2 a set of variables capturing non-COVID-19-related health aspects that might predispose people to getting infected and/or might increase the risk of complications when infected with SARS-Cov-2, i.e. chronic obstructive pulmonary disease (COPD), diabetes and smoking. In addition, we add the mortality rates for respiratory and cardiovascular causes and diabetes. This dataset also includes physician density and (where available) the number of beds in intensive care and/or reanimation units available in hospitals at NUTS2 level.

**Table 1:** COVID-19 variables – COVID-19.

Variable name	Variable description	Unit	Spatial range	Time range
COVID-19_D	Number of deaths due to COVID-19	Number (aggregated)	NUTS3	March-Aug. 2020
COVID-19_CCONF	COVID-19 cases confirmed	Number (aggregated)	NUTS3	March-Aug. 2020
COVID-19_TESTS	Number of tests taken for COVID-19	Number (aggregated)	NUTS3	March-Aug. 2020

Note: The sources of these COVID-19 variables are given in the database [1].

**Table 2:** Health variables (disease incidence, mortality, health behaviors and medical infrastructure).

Variable name	Variable description	Unit	Spatial resolution	Year of data
COPD%	Percentage of population with Chronic Obstructive Pulmonary disease	Percentage	NUTS2	2018
Diabetes%	Percentage of population with diabetes	Percentage	NUTS2	2018
Smokers%	Percentage of population that smoke	Percentage	NUTS2	2018
Respiratory disease mortality	Mortality rate at 100.000 persons for deaths attributable to respiratory disease	Rate at 100.000 inhabitants	NUTS2	2018
Diabetes mortality	Mortality rate at 100.000 persons for deaths attributable to diabetes	Rate at 100.000 inhabitants	NUTS2	2018
Cardiovascular dis mortality	Mortality rate at 100.000 persons for death attributable to cardiovascular disease	Rate at 100.000 inhabitants	NUTS2	2018
BEDS_Intcare/reanim	Number of beds in intensive care/reanimation	Number (aggregated)	NUTS3	2018
D_MEDICAL	Physician density	Doctors/100.000 inhabitants	NUTS2	2018

Note: The sources of these health variables are given in the database [1].

Table 3 describes the socio-demographic and economic data available at NUTS3 level for all European countries (source: Eurostat). This data comprises population density, the population growth, and the surface area of the region. In addition, we provide the population split into five age groups, as well as the percentage of the population of aged people above 60 years old and the percentage of females and males in the population. We also include variables capturing the number of households and dwellings at NUTS3 level. The economic data refers to the unemployment rate at NUTS2 level and the nightlight intensity, for which we have collected its average from the year 2016 at NUTS3.

**Table 3:** Socio-demographic and economic variables.

Variable name	Variable description	Unit	Spatial range	Year of data
POPULATION	Population	Number (aggregated)	NUTS3	2019
POP_DENS	Density population	p/km2	NUTS3	2019
POP>=60	Population over 60 years old	Number (aggregated)	NUTS3	2019
%POP>=60	Percentage of population over 60 years old	Percentage	NUTS3	2019
FEMALES	Population of females	Number (aggregated)	NUTS3	2019
%FEMALES	Percentage of population of females	Percentage	NUTS3	2019
POP 0–14	Population 0–14 years old	Number (aggregated)	NUTS3	2019
POP 15–29	Population 15–29 years old	Number (aggregated)	NUTS3	2019
POP 30–44	Population 30–44 years old	Number (aggregated)	NUTS3	2019
POP 45–59	Population 45–59 years old	Number (aggregated)	NUTS3	2019
POP_GROWTH	Population growth	Percentage	NUTS3	2019
HOUSEHOLDS	Number of households	Number (aggregated)	NUTS3	2019
DWELLINGS	Number of dwellings	Number (aggregated)	NUTS3	2019
UNEM_R	Unemployment rate	Percentage	NUTS2	2018
NTL	Night Light Intensity average	Between 1 and 61	0.1°	2016
SURFACE AREA	The surface area of each region	km²	NUTS3	2019

Note: The sources of these socioeconomic variables are given in the database [1].

Table 4 includes the environmental variables. For these variables, we have collected the annual average over a period of 16 years that were averaged and aggregated at the NUTS3 level. Table 5 refers to the variables tracking the public policies put in place by authorities to mitigate the spread of the virus (i.e. lockdown measures). We have calculated the number of days since the first case reported until the first day of lockdown as well as the duration of lockdown in each country. Furthermore, we add a variable describing the lockdown severity in each country. All tables include three more variables: COUNTRY, CODE_COUNTRY, NUTS3, CODE_NUTS3. COUNTRY represents the name of the country and NUTS3 the sub-regions, the CODE_COUNTRY is the letter code of each country (e.g., LUX for Luxembourg), and the NUTS3_CODE is the classification code for each sub-region NUTS3. However, in some open sources for COVID-19, the data was available only at NUTS2 level; thus, we include this data as well as at NUTS2.

**Table 4:** Environmental variables.

Variable name	Variable description	Unit	Spatial range	Years of data
NO_2_	Annual mean of NO_2_	Billions of nitrogen dioxide (ppm)	0.1-degree	1996–2012 and 2018–2020
WIND	Annual mean of Wind speed	m/s	0.1°	1996–2012
TEMP	Annual mean of Temperature over 12 years	°C	0.1°	1996–2012
PM_2.5_	Annual mean of PM_2.5_	µg/m3	0.1°	1996–2012
PRESSURE	Water-vapor pressure	kPa	0.1°	1996–2012
PRECIPITATION	Precipitation average	mm	0.1°	1996–2012
SOLAR_RAD	Solar Radiation average	kJ/m2/day	0.1°	1996–2012
LAI	Leaf Area Index average	Values between 0 and 7	0.1°	1999- 06/2020

Note: The spatial resolution refers to the resolution at which the dataset was downloaded. Our dataset contains the same variable aggregated at NUTS3 level and the data sources of these variables are given in the supplementary data are available dataset [1].

**Fig. 1:**
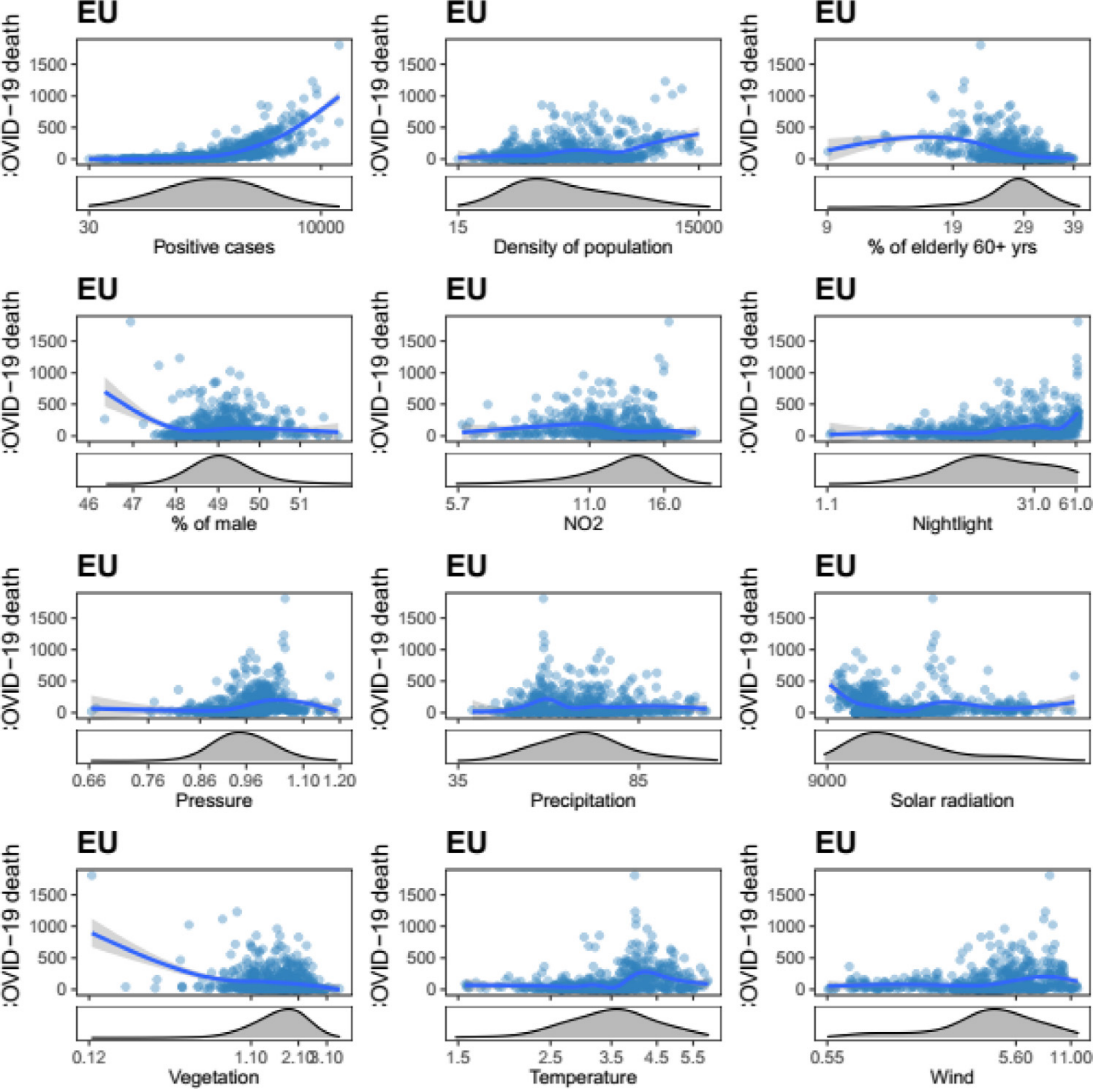
Key variables included in the dataset, given as an average value at NUTS3 across Europe.

**Table 5:** Public policy related to COVID19 variables.

Variable name	Variable description	Unit	Spatial range	Year of data
DURATION _LD	Duration of Lock-Down in days	Number of days	NUTS1	2020
Lag_1stCase _LD	Lag period from the first case until the first day of lockdown decision.	Number of days	NUTS1	2020
Severity _LD	Lockdown severity	0 - No measures 1 - Recommended not to leave the house 2 - Required not to leave the house with exceptions for daily exercises 3 - Required not to leave the house with minimal exceptions.	NUTS1	2020

In Fig. 1, we present the relationship of a sample of variables of the dataset with COVID-19 mortality and positive cases. This figure is given as an example to illustrate the potential use and usefulness of this dataset.

## 2 Experimental Design, Material and Methods

**Fig. 2:**
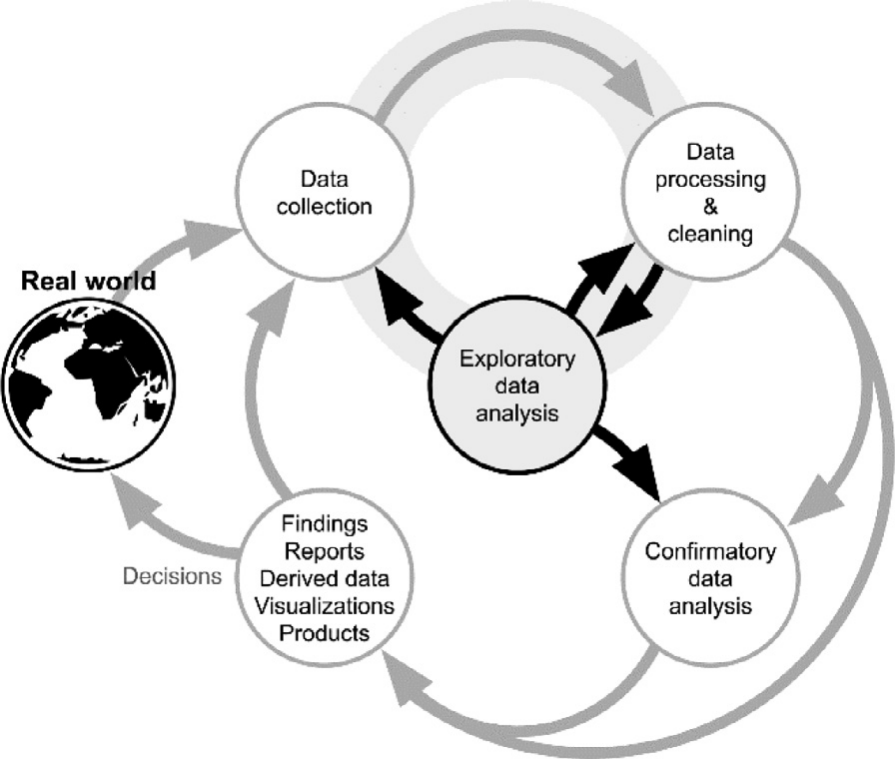
Workflow of the process of data collection and processing (adapted from [7]).

Due to the outbreak of the novel coronavirus pandemic at the beginning of 2020, several countries around the world developed dashboards [4,5] and open data sources [6] that provide open access to COVID-19 data in real time and/or over time (i.e., daily, weekly, monthly). These open sources have the scope of informing the population of the status of the pandemic and help researchers in understanding the impact of the virus on our surroundings. However, generally COVID-19 dashboards provide aggregated data at the NUTS1 level and rarely at the sub-national levels (those from governmental agencies). To overcome this limitation we have collected COVID-19 data from multiple sources at the lower administrative possible scale (NUTS2–3) and compiled them in one place. In order to build this dataset we followed the workflow described in Fig. 2 [7]. This workflow is composed by several processes: data collection, processing/cleaning, analysis, and visualization. The resulting dataset is ready-to-use by a large community of researchers in a wide range of applications [1]. It contains 35 socio-demographic, economic, public policy, health, air pollution and environmental variables that can help researchers, practitioners, authorities, and those interested in this subject.

**Fig. 3:**
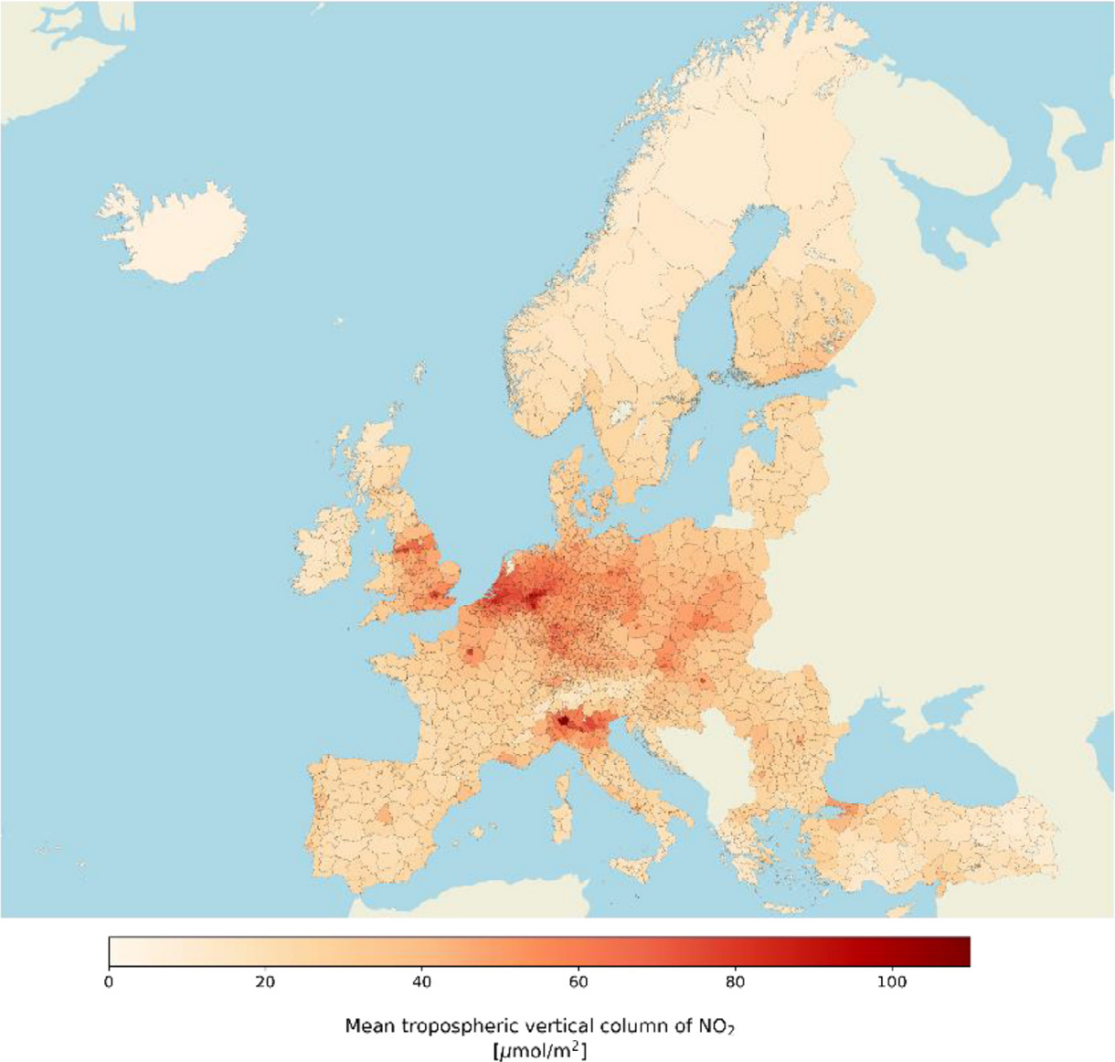
Distribution of monthly average NO_2_ concentration across the entire Europe (March 2020).

**Fig. 4:**
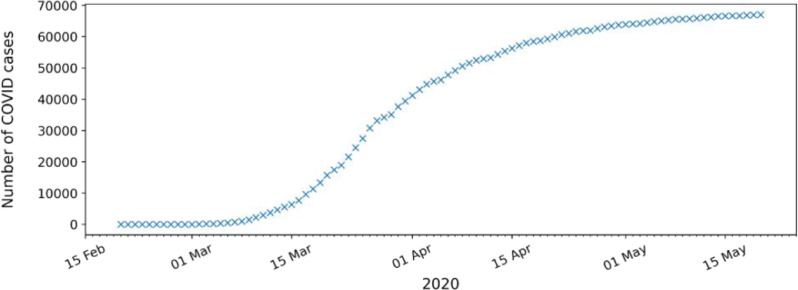
COVID-19 cases over time (Feb.-May 2020) in Madrid NUTS3.

To visualize all the collected data at NUTS3 level (with both static and dynamic component), a web-based dashboard application was developed [2]. This application allows automatic processing of spatial *Raster* and *Vector* datasets, to get relevant statistics (i.e., mean, minimum, maximum, and standard deviation). This application also shows interactively the number of COVID-19 mortality and positive cases, simultaneously. The user is able to set the region of interest (i.e., country), the NUTS level (i.e., NUTS1-2-3), type of pollutant (i.e., NO_2_), the year and the desired statistics. Then, a choropleth map is generated, accompanied by COVID-19 cases evolution chart of the selected area. As an example, Fig. 3 shows the distribution of NO_2_ across the entire Europe during March 2020 at the NUTS 3 level [8]. In addition, the dashboard generates charts showing temporary changes of COVID-19 mortality and positive cases, such as the example in Fig. 4 that shows the daily variation of COVID-19 positive cases in Madrid NUTS3.

## Ethics Statement

None.

## CRediT Author Statement

**Hichem Omrani:** Conceptualization, Methodology, Supervision, Project administration, Funding acquisition, Writing - Reviewing and Editing; **Madalina Modroiu:** Data curation, Investigation, Writing - Original draft preparation; **Javier Lenzi:** Investigation, Formal analysis, Writing - Reviewing and Editing; **Bilel Omrani:** Software, Visualization, Investigation; **Zied Said:** Software, Visualization; **Marc Suhrcke:** Validation, Writing - Reviewing and Editing; **Anastase Tchicaya:** Validation, Writing-Reviewing and Editing; **Nhien Nguyen:** Writing - Reviewing and Editing; **Benoit Parmentier:** Validation, Writing-Reviewing and Editing.

## Declaration of Competing Interest

The authors declare that they have no competing financial interests or personal relationships, which could have influenced the work reported in this article.

## Appendix Supplementary materials

Image, application 1
